# Clarifying the Concept of Adherence to eHealth Technology: Systematic Review on When Usage Becomes Adherence

**DOI:** 10.2196/jmir.8578

**Published:** 2017-12-06

**Authors:** Floor Sieverink, Saskia M Kelders, Julia EWC van Gemert-Pijnen

**Affiliations:** ^1^ Centre for eHealth and Wellbeing Research Department of Psychology, Health and Technology University of Twente Enschede Netherlands; ^2^ Optentia Research Focus Area North-West University Vanderbijlpark South Africa

**Keywords:** adherence, eHealth, systematic review

## Abstract

**Background:**

In electronic health (eHealth) evaluations, there is increasing attention for studying the actual usage of a technology in relation to the outcomes found, often by studying the adherence to the technology. On the basis of the definition of adherence, we suggest that the following three elements are necessary to determine adherence to eHealth technology: (1) the ability to measure the usage behavior of individuals; (2) an operationalization of intended use; and (3) an empirical, theoretical, or rational justification of the intended use. However, to date, little is known on how to operationalize the intended usage of and the adherence to different types of eHealth technology.

**Objective:**

The study aimed to improve eHealth evaluations by gaining insight into when, how, and by whom the concept of adherence has been used in previous eHealth evaluations and finding a concise way to operationalize adherence to and intended use of different eHealth technologies.

**Methods:**

A systematic review of eHealth evaluations was conducted to gain insight into how the use of the technology was measured, how adherence to different types of technologies was operationalized, and if and how the intended use of the technology was justified. Differences in variables between the use of the technology and the operationalization of adherence were calculated using a chi-square test of independence.

**Results:**

In total, 62 studies were included in this review. In 34 studies, adherence was operationalized as “the more use, the better,” whereas 28 studies described a threshold for intended use of the technology as well. Out of these 28, only 6 reported a justification for the intended use. The proportion of evaluations of mental health technologies reporting a justified operationalization of intended use is lagging behind compared with evaluations of lifestyle and chronic care technologies. The results indicated that a justification of intended use does not require extra measurements to determine adherence to the technology.

**Conclusions:**

The results of this review showed that to date, justifications for intended use are often missing in evaluations of adherence. Evidently, it is not always possible to estimate the intended use of a technology. However, such measures do not meet the definition of adherence and should therefore be referred to as the actual usage of the technology. Therefore, it can be concluded that adherence to eHealth technology is an underdeveloped and often improperly used concept in the existing body of literature. When defining the intended use of a technology and selecting valid measures for adherence, the goal or the assumed working mechanisms should be leading. Adherence can then be standardized, which will improve the comparison of adherence rates to different technologies with the same goal and will provide insight into how adherence to different elements contributed to the outcomes.

## Introduction

### Adherence and Attrition

One of the main goals of electronic health (eHealth) evaluations is to gain insight into the effects of technology on outcomes such as quality of life, health-related outcomes (eg, glycemic control, weight loss), or psychological outcomes (eg, depressive complaints, anxiety). However, many eHealth evaluations report no or limited positive effects [1-5]. There is strong evidence that this is often related to participants not using technologies in the desired way. For every technology, a proportion of the users will not use the intervention at all, will stop using the technology after a period, or will not use the available elements of the technology as intended [1,6-8].

To gain more insight into this phenomenon, Eysenbach made a plea back in 2005 for reporting the levels of nonusage attrition, or the extent to which individuals stop using the technology [9]. On the other hand, understanding adherence, or how actual usage of the technology may have influenced the outcomes, might be just as important [6]. The term adherence is rooted in the pharmaceutical industry, and according to the World Health Organization’s (WHO) definition, it refers to “the extent to which a person’s behaviour – taking medication, following a diet, and/or executing lifestyle changes, corresponds with agreed recommendations from a health care provider *”* [10].

### Adherence to eHealth Technologies

For eHealth technologies, several definitions for adherence can be identified in the existing literature. For example, Christensen et al defined adherence as “the degree to which individuals experience the content of the Internet intervention” [11]. However, the concept of “following the prescribed recommendations” (as implied by the WHO’s definition) is missing from this definition. Therefore, Donkin et al referred to adherence as “the degree to which the user followed the program as it was designed” [6]. In accordance with the WHO definition of adherence, this definition contains the concept of intended use, or “the extent to which individuals should experience the content to derive maximum benefit from the intervention, as defined or implied by its creators” [1]. According to these definitions, the intended use is thus the minimum use to establish adherence.

Although adherence is related to other measures such as engagement or nonusage attrition, these terms do not refer to the same or inverse concepts. After all, not using the technology as defined or implied by its creators does not necessarily mean that a participant is not using the technology at all (as implied by the definition of nonusage attrition) [9]. Moreover, definitions of engagement usually incorporate the more subjective attributes of challenge, positive affect, endurability, and aesthetic and sensory appeals [12], whereas adherence is mostly based on measures for usage behavior.

### Determining Adherence

There is now increasing attention for studying the adherence rates and reasons for nonadherence in eHealth evaluations. However, it still can be a challenge to operationalize the intended use for individual eHealth technologies in a certain context. In the pharmaceutical industry, the intended use (ie, agreed recommendations) is mostly based on the observed or rationalized working mechanisms and the dose-response curves of the medication for a certain condition. As a result, the dosage of one particular medication can vary depending on (the severity of) the condition and the patient’s characteristics (eg, age, gender, or weight).

This is in contrast with many prior eHealth studies, which often assume that all users should experience all of the elements of a technology to obtain effects, and in which adherence is thus often operationalized as using everything the technology offers. However, a technology can be designed for multiple target groups and, depending on the individual user goals and the desired outcomes, technology can be used in many different ways in terms of the features that are used, as well as the frequency, time, and place of use [13,14]. Furthermore, the amount of use that is needed to obtain the desired outcomes may vary a lot across different user groups [6]. This implies that users do not always have to experience all of the available elements of a technology or have to use the same elements because usage goals may differ across users as well. Moreover, individuals may also stop using the technology because they have reached their personal goals (early completers or e-attainers) [11,15], and nonusage dropout is thus not always a consequence of losing interest (as stated by Eysenbach) [9].

To summarize, based on the definition of adherence, we suggest that the following three elements are necessary to determine adherence to eHealth technology: (1) the ability to measure the usage behavior of individuals; (2) an operationalization of intended use; and (3) an empirical, theoretical, or rational justification of the intended use. However, to date, little is known about how to operationalize the intended usage of and thus the adherence to different types of eHealth technology. Many systematic reviews gaining insight into adherence to eHealth technology focus on the extent to which individuals use different types of technology and what the reasons for nonadherence are, without a proper operationalization of intended use and adherence [1,6,11,16,17]. These reviews therefore fail to provide insight into how adherence and intended use have been operationalized.

### The Goal of the Review

The goal of this systematic review was to improve evaluations of eHealth technologies by gaining insight into when, by whom, and how the concept of adherence has been used in previous eHealth evaluations, and finding a concise way to operationalize the adherence to and the intended use of different eHealth technologies. We do this by providing insight into how the usage of the technology was measured across previous studies; how adherence to different types of technologies (eg, structured interventions, patients platforms) was operationalized; and if and how the intended use of eHealth technologies has been justified with theory, evidence, or rationale.

## Methods

### Search Strategy

A literature search was conducted using the Scopus, Web of Science, ScienceDirect, and PsycINFO databases. A combination of the constructs “technology,” “intervention,” “adherence,” and “health” was used. To ensure sufficient coverage of each construct, we used different keywords for every construct (see Multimedia Appendix 1). We excluded other usage-related concepts (eg, nonusage attrition or engagement) because these do not refer to the same concept.

### Eligibility Criteria

All articles that met the following criteria were included in the review: (1) it involved health-related technology (Web-based technologies, apps, wearables, or technologies provided via other devices); (2) the technology was intended to be used more than once by the patient or client; (3) the article described a primary study or a protocol for a primary study that included objective, quantifiable measurements, and an operationalization of adherence to the technology; (4) the study was published in English; and (5) the study was peer-reviewed and published.

Articles were excluded in the following situations: (1) adherence was defined as adhering to offline treatment or as a measure for following a study protocol, (2) the technology studied was only used as a tool for exchanging information without the possibility for further interaction with the system (eg, telemonitoring only, sending or receiving messages like SMS [short message service] interventions, or in chat rooms), and (3) the article was a conference abstract or full text was not available.

### Study Selection

The selection of studies was completed in three steps. First, all titles were screened by two authors (FS and SK) to exclude the records that clearly indicated a study outside the scope of this review (eg, medication adherence). Second, the abstracts of the articles initially deemed relevant were screened for eligibility by the same authors. During this process of title and abstract screening, studies were included in the next step if they were deemed eligible by at least one of the reviewers.

Third, the full texts of all remaining publications were checked for inclusion by FS, and the final selection was discussed by FS, SK, and LvG. Disagreements regarding the inclusion of full texts were discussed until consensus was reached.

### Data Collection and Analysis

The required information for all included technologies and studies was coded by FS using a data extraction form. The information that was extracted from each article is presented in Textbox 1.

On the basis of the extracted information, the operationalizations for adherence in every study were categorized. An overview of these categories is provided in Table 1.

All the data on each study were entered in SPSS version 24.0 (IBM Corporation, Somers, NY, USA). Each was treated as a separate case. The results are categorized based on the use of the technology (structured, hybrid, and unstructured) and the categorization of adherence operationalizations (Category A, B, and C). Descriptive data for the different categories were calculated using SPSS. Differences in variables between the use of the technology and the operationalization of adherence were calculated using a chi-square test of independence. When the observed counts were below the expected counts, a Monte Carlo correction was applied. We used an alpha level of .05 for all statistical tests.

**Information extracted from the included articles.**

1. *General information* regarding the authors, affiliation, country, year, and journal of publication.
2. *The name of the technology*: when no name was reported, the name was indicated as “N/A.”
3. *The type of technology or the device* **:** for example, Web-based, mobile phone apps, wearable, or other devices for monitoring.
4. *Type of use (structured, hybrid, or unstructured)*: “Structured use” was assigned to technologies consisting entirely of separate modules or lessons that users had to complete before moving on to the next [6]. “Free use” was assigned to technologies that consisted of different elements that users could then use at their own convenience (eg, a personal health record containing a diary, educational material, and a messaging function; or a wearable connected to a mobile phone app to gain insight into something like activity levels). “Hybrid use” was assigned to technologies with a fixed core, supplemented with other components for free use.
5. *The health care field targeted with the technology*, distinguishing between mental health (eg, targeting depressive symptoms or anxiety), chronic conditions (eg, self-management support for patients with type 1 diabetes mellitus), or lifestyle technologies (eg, losing weight, improving physical activity, or quitting smoking). These categories were assigned depending on the technology’s goal, meaning that an intervention to support patients with chronic conditions maintaining a healthy lifestyle is seen as a lifestyle technology.
6. *The variables that were used to assess adherence*, such as the number of logins, the number of different days that users used the technology, the time spent on the technology, the number of modules or lessons started or completed, and the number of different elements that were accessed or used.
7. *The intended use* of the technology.
8. *Whether the described intended use was justified*, for example, using theory, evidence, or rationale.

**Table 1:** Categorization of adherence operationalizations.

Category	Explanation
Category A	Assigned when adherence was operationalized in terms of “the more usage, the better.” Category A operationalizations do not include an operationalization of intended use, and therefore do not comply with the definition of adherence.
Category B	Assigned when the intended use of a technology was provided without justification (eg, “a user is adherent when logging in at least once a week for three subsequent weeks”).
Category C	Assigned when the intended use of the technology was provided *and* justified using theory, evidence, or rationale (eg, “we know from previous research that users benefit the most from the technology when finishing module 4, so a user is adherent once module 4 is completed”).

## Results

### Study Selection

A total of 7005 studies were identified via the search. After screening of the titles, abstracts, and full texts, 62 full texts were included in this review. An overview of these articles is presented in Multimedia Appendix 2.

**Figure 1:**
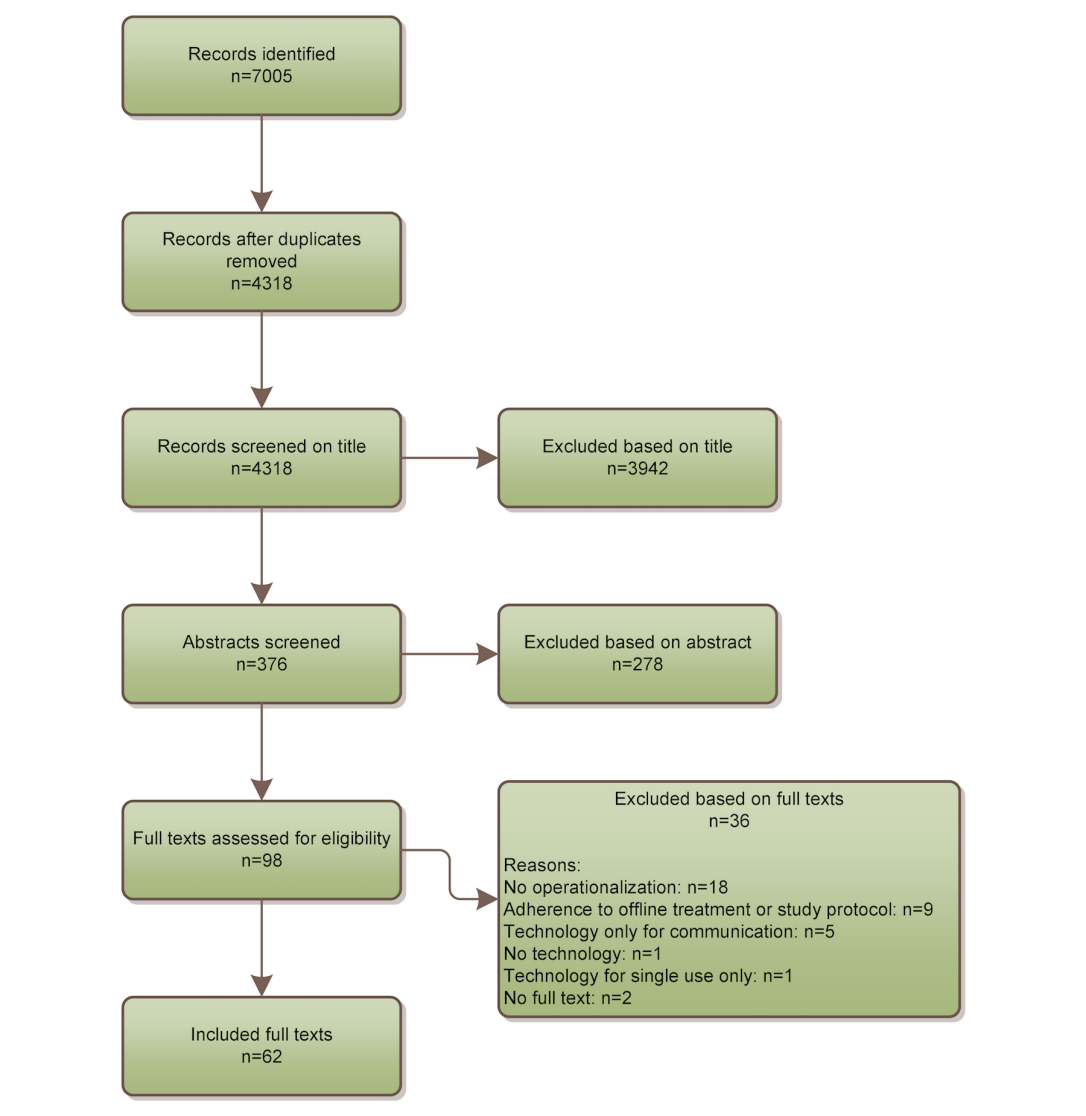
Flowchart of full text selection.

In total, 36 articles were excluded during the full-text screening phase (Figure 1). Most full texts (n=18) were excluded because they did not include objective, quantifiable measurements, and an operationalization of adherence to the technology (12 primary studies and 6 viewpoint papers). Other reasons for exclusion are presented in Figure 1.

**Figure 2:**
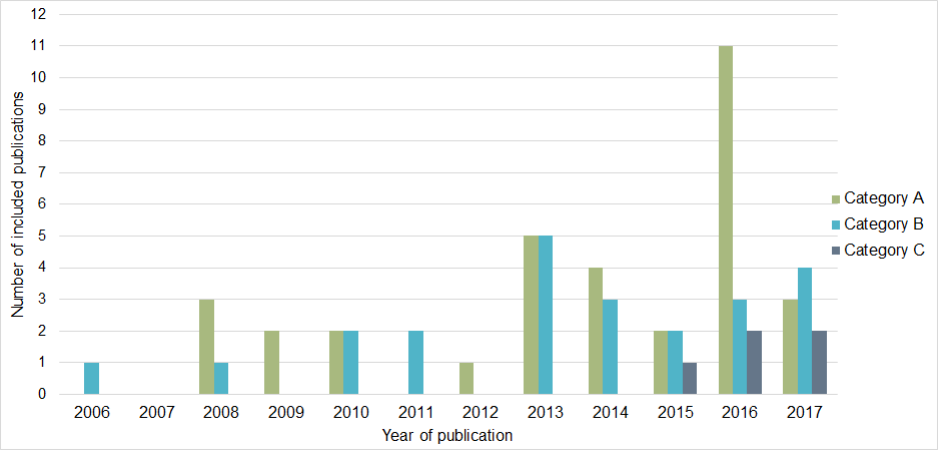
The number of studies reporting Category A, B, and C operationalizations per publication year.

All included articles were published in 2006 or later, and more articles published in recent years were included overall (Figure 2). The first authors are mostly affiliated in the United States of America (n=15), Australia (n=10), and the Netherlands (n=8) (Table 2). In total, 24 of the studies were published in the *Journal of Medical Internet Research* or its sister journals.

### Technology Characteristics

Table 3 provides an overview of the technologies that are subjects of the included studies. The technologies described in most of the articles are Web-based (51/62). Furthermore, five are smartphone apps and five are Web-based or smartphone technologies combined with other devices such as wearables. Almost half of the technologies (29/62) were structured technologies, 18 were unstructured technologies, and 15 had a hybrid nature.

Half of all included articles reported adherence to mental health technologies. Most of these technologies targeted depression (n=8) and anxiety disorders (n=5), some of the latter also in combination with depression (n=3). Other mental health technologies targeted postdisaster mental health distress (n=3); cancer-related distress (n=3); general stress management (n=2); eating pathology (n=2); or insomnia, erectile dysfunction, bipolar disorders, mindfulness, and cognitive training (all n=1). Eighteen of these technologies were based on cognitive behavioral therapy. Most of the structured technologies (17/29) and almost all hybrid technologies (12/15) were aimed at improving mental health (*P*<.001).

A total of 25 technologies were aimed at supporting a healthy lifestyle, more specifically smoking cessation (n=7); improving physical activity (n=7); weight loss (n=5); alcohol cessation (n=3); general health promotion (n=2); or healthy eating (n=1). Six technologies were aimed at self-management support for patients with chronic diseases (diabetes [n=3], inflammatory bowel disease [n=1], hypertension [n=1], or surgical site infections [n=1]). Most of the unstructured technologies were aimed at lifestyle support (13/18).

For all technologies, adherence was mostly operationalized using measures regarding the number of modules or lessons completed and the number of different days, weeks, or months that people used the technology. Adherence to unstructured technologies was mostly operationalized using the number of logins or sessions (*P*=.03), the number of features accessed or used (*P*<.001), and the time spent using the technology (*P*<.001). Adherence to structured technologies was most often operationalized using the number of modules or lessons completed (*P*<.001).

### Operationalization of the Adherence Definition

Out of the 62 included articles, 34 reported adherence only in terms of how often the technology was used (Category A operationalization) [7,18-50]. In 23 studies, the intended usage was described as well (Category B operationalization) [51-73], and 5 studies reported the intended usage with a justification for this threshold (Category C operationalization) [74-78]. The number of publications reporting Category C operationalizations has increased since 2015 (Figure 2).

Table 4 provides an overview of the characteristics of the studies for adherence category. Although no significant differences were found, we were still able to identify some interesting patterns. Overall, the data show that 20 out of 31 technologies for mental health contain a Category A operationalization, whereas Category C operationalizations are more equally distributed over the three health care fields. Furthermore, 48 out of 56 Category A and Category B operationalizations are for Web-based technologies (whether or not in combination with other devices), whereas a third of the Category C operationalizations are also for smartphone technologies.

Most Category A operationalizations contain a measure for the number of modules that the users accessed or completed (19/34) and the time spent on the technology and number of features accessed and used (both 11/34). Most Category B operationalizations contain the number of days, weeks, or months that people used the technology (11/23), the number of accessed or completed modules (8/23), and the number of logins or sessions (4/23).

Category C definitions are mostly based on the number of accessed or completed modules (3/5 operationalizations). The number of logins and the number of days, weeks, or months that people used the technology were used in 2 out of 5 operationalizations.

Most operationalizations of adherence are based on a maximum of two different measures, regardless of the category (49/62). Ten out of 13 operationalizations that feature 3 or more measures are categorized as Category A.

The included Category C operationalizations provided justification in various ways. Reinwand et al asked all participants to complete a questionnaire to assess to what extent their lifestyle met the Dutch guidelines for healthy eating, drinking alcohol, physical activity, etc [74]. Recommendations for the use of corresponding elements of the technology were then made based on the outcomes of the assessment and, in turn, adherence was defined as using the technology in accordance with these recommendations. In the study by Zeng et al, the technology consisted of different elements that were all evaluated as effective in other studies [75]. Users were considered adherent if they used all elements. Beatty et al considered a user to be highly adherent when a therapeutic dose of 66% of the intervention was received [76]. This threshold for a therapeutic dose was obtained from previous studies. In the study by Mertens et al, technology use was represented in relation to medication use, and users were therefore seen as adherent when the technology was used in accordance with the recommendations for medication use [77]. Carolan et al describe a protocol for a study to be conducted to understand the optimum adherence to the technology in relation to the outcomes [78].

**Table 2:** Country of affiliation of the first authors of all included articles.

Country	Number of included articles
United States of America	15
Australia	10
The Netherlands	8
Sweden	5
United Kingdom	5
Canada	3
Germany	3
Switzerland	3
China	2
Norway	2
Austria	1
Denmark	1
Finland	1
Ireland	1
Portugal	1
Spain	1

**Table 3:** Characteristics of structured, unstructured, and hybrid technologies and their operationalizations of adherence.

Characteristics		Structured (n=29)	Unstructured (n=18)	Hybrid (n=15)
		n (%)	n (%)	n (%)
**Health care field**^a^				
	Mental health (n=31)	17 (59)	2 (11)	12 (80)
	Lifestyle (n=25)	9 (31)	13 (72)	3 (20)
	Chronic care (n=6)	3 (10)	3 (17)	0 (0)
**Device**				
	Web-based (n=51)	25 (86)	13 (72)	13 (87)
	Smartphone app (n=6)	3 (10)	2 (11)	1 (7)
	Web-based or smartphone with wearable (n=2)	0 (0)	1 (6)	1 (7)
	Web-based or smartphone with wearable and monitoring device (n=3)	1 (3)	2 (11)	0 (0)
**Level of adherence definition**				
	Category A (n=34)	16 (55)	10 (56)	8 (53)
	Category B (n=23)	11 (38)	7 (39)	5 (33)
	Category C (n=5)	2 (7)	1 (6)	2 (13)
**Number of measures**^a^				
	1 (n=32)	19 (66)	4 (22)	9 (60)
	2 (n=17)	8 (28)	7 (39)	2 (13)
	3 (n=7)	1 (3)	3 (17)	3 (20)
	4 or more (n=6)	1 (3)	4 (22)	1 (7)
**Measures of adherence**				
	Number of logins/number of sessions (n=14)^a^	3 (10)	8 (44)	3 (20)
	Number of modules/number of lessons completed (n=30)^a^	17 (59)	2 (11)	11 (73)
	Number of features accessed/used (n=16)^a^	5 (17)	9 (50)	2 (13)
	Number of exercises completed (n=9)	6 (21)	2 (11)	1 (7)
	Number of pages viewed (n=11)	2 (7)	6 (33)	3 (20)
	Number of days/weeks/months (n=19)^a^	8 (28)	7 (39)	4 (27)
	Time spent (n=14)^a^	2 (7)	9 (50)	3 (20)

^a^*P*<.05.

**Table 4:** Characteristics of Category A, B, and C definitions.

Characteristics		Category A (n=34)	Category B (n=23)	Category C (n=5)
		n (%)	n (%)	n (%)
**Health care field**				
	Mental health (n=31)	20 (59)	9 (39)	2 (40)
	Lifestyle (n=25)	13 (38)	10 (44)	2 (40)
	Chronic care (n=6)	1 (3)	4 (17)	1 (40)
**Device**				
	Web-based (n=51)	31 (91)	17 (74)	3 (60)
	Smartphone app (n=6)	1 (3)	3 (13)	2 (40)
	Web-based or smartphone with wearable (n=2)	1 (3)	1 (4)	0 (0)
	Web-based or smartphone with wearable and monitoring device (n=3)	1 (3)	2 (9)	0 (0)
**Type of use**				
	Structured (n=29)	16 (47)	11 (48)	2 (40)
	Unstructured (n=18)	10 (29)	7 (30)	1 (20)
	Hybrid (n=15)	8 (24)	5 (22)	2 (40)
**Measures of adherence**				
	Number of logins/number of sessions (n=14)	8 (24)	4 (17)	2 (40)
	Number of modules/number of lessons completed (n=30)	19 (56)	8 (35)	3 (60)
	Number of features accessed/used (n=16)	11 (32)	4 (17)	1 (20)
	Number of exercises completed (n=9)	6 (18)	3 (13)	0 (0)
	Number of pages viewed (n=11)	9 (27)	1 (4)	1 (20)
	Number of days/weeks/months (n=19)^a^	6 (18)	11 (48)	2 (40)
	Time spent (n=14)	11 (32)	2 (9)	1 (17)
**Number of measures**				
	1 (n=32)	13 (38)	16 (70)	3 (60)
	2 (n=17)	11 (32)	5 (22)	1 (20)
	3 (n=7)	6 (18)	1 (4)	0 (0)
	4 or more (n=6)	4 (12)	1 (4)	1 (20)

^a^*P*<.05.

## Discussion

### Aim of this Review

In this systematic review, we have sought to gain insight into how the concept of adherence has been used in previous eHealth evaluations. In line with the definitions for adherence and intended use maintained by the WHO [10] and Kelders [1], we reviewed not only how usage was measured but also if and how intended use of eHealth technologies was operationalized and justified using theory, evidence, or rationale.

### Principal Findings

We included 62 studies in this review, all published after 2005. The majority of the technologies described in these studies were structured or hybrid Web-based interventions targeting mental health (mostly cognitive behavioral therapy [CBT] interventions) or unstructured technologies for lifestyle support.

We observed a growing number of studies that studied adherence to eHealth technologies since Eysenbach’s plea for reporting the levels of nonusage attrition with eHealth technology in 2005 [9]. Although the “prescribed recommendations” or the intended use of a technology form an important element of the definition of adherence [10], and although there is evidence that users do not always have to complete an intervention to experience effects [6,11,13-15], half of all operationalizations are based on the assumption of “the more use, the better” and do not include a threshold for intended use. Sometimes, we do not know (yet) what the intended use of a technology is, or defining the intended use is not necessary to answer the research question(s) of the study. Then, a Category A operationalization suffices for answering the research questions. However, a Category A operationalization only refers to the actual usage of a technology without comparing it with its intended usage. According to the definitions used, they should therefore not be referred to as adherence.

When the intended use for the technology was reported, only a minority of all included studies featured justified Category C operationalizations, making the comparison of adherence across different eHealth technologies more complicated. However, we were still able to observe a small increase in Category C operationalizations since 2015.

Remarkably, the proportion of evaluations of mental health technologies reporting a justified (Category C) operationalization of intended use is lagging behind compared with evaluations of lifestyle and chronic care technologies. This is unexpected because the majority of mental health technologies is based on (principles of) CBT, which is the most studied treatment for depression and has proven effective in many studies [79]. However, a meta-analysis of Van Ballegooijen et al revealed that participants complete approximately 84% of their CBT program in both offline and online treatment. Although a longer treatment duration is associated with better effects [80], this still implies that users do not necessarily need to finish the complete program to experience reduction of their complaints, and that there should be a threshold for intended use. As such, it seems that this knowledge could be used to define and substantiate the intended use of a CBT technology, but in the studies included in this review, this notion has not been put into practice yet.

We were also able to observe some interesting patterns in the composition of measures for adherence and intended use. When the operationalization of adherence consists of a combination of four or more measures, it is most likely a Category A operationalization, whereas most Category C operationalizations consist of one or two measures. This implies that for many Category A operationalizations, a scattershot approach was used when it comes to measuring adherence. In contrast, the results indicated that justifications of intended use are often based on the goal of the technology and/or the assumed working mechanisms, leading to more focused operationalizations that do not require additional measurements to evaluate adherence to the technology. In other words, more measures are not necessarily the key to knowledge if they are not sufficiently specific.

No significant differences could be found between the kinds of measures that are used for all three levels of operationalizations. Category A operationalizations most often contain the number of modules that a user started or completed, the number of features accessed or used, and the time spent online. This seems obvious, as this level of operationalization is mostly used for structured or hybrid mental health interventions consisting of different modules that users have to follow. Category C or justified operationalizations are more often defined by the number of days, weeks, or months that the technology is used by people. This can be explained by way of the finding that Category C operationalizations are used for a large proportion of unstructured and hybrid technologies. As people are able to use the features of these technologies more or less at their own convenience, the development of use over time would probably provide more information regarding adherence than use of the technology’s content at fixed points in time only.

### Implications and Recommendations

An important reason for the lack of justifications for the intended use of eHealth technologies might be that there is a lack of knowledge regarding the working mechanisms of technology-based applications [8]. However, the included Category C operationalizations did show that knowledge of the working mechanisms of the technology is not a prerequisite for defining the intended use. After all, the intended use or the “therapeutic dose” can be justified just as well using existing guidelines for healthy living and medication use [74,77] or using previous research regarding other technologies [75,76]. Moreover, the intended use has also been operationalized by linking the (positive) outcomes of individual users to their usage patterns to find the most effective patterns [78].

The fact that we did not find a justification of intended use based on existing models for behavior change was unexpected. For instance, Kaushal and Rhodes found that exercising for at least four times per week for 6 weeks is a minimal requirement for establishing exercise habits [81]. These kind of findings can also be used for determining intended use; for example, a user of a technology to improve physical activity is adherent when using (specific elements of) the technology at least four times a week and 6 weeks in a row.

Another example comes from Kelders et al who found that a group of users dropped out from an intervention for reducing depressive complaints after a lesson that focused on applying newly acquired skills in practice, as doing so can be confrontational [56]. However, following this lesson can also be seen as an important precondition for gaining effects from using the intervention. The intended use of this intervention could thus be operationalized as following the intervention until that critical lesson is completed at the very least. An important aspect of operationalizing the intended use of a technology is therefore to keep the goal of the technology and the desired outcomes in mind. What use is necessary at minimum to reach that goal (eg, to experience certain effects or establish new skills and habits), and how can we translate this into measures for adherence?

Although it has previously been suggested that a combination of a range of different variables for technology usage provides a more meaningful measure of adherence [6], the results of this review show that a limited but deliberate set of only one or two different measures in accordance with the goal of the technology can also be used for operationalizing intended use. At the moment, eHealth evaluations often fail to demonstrate the dose-response relationship (the usage that is minimally needed to experience certain effects) or simply define it as “the more use, the better.” However, the results of this review indicate that Category A and B operationalizations of adherence often do not take the characteristics of the technology (eg, goal, persuasiveness, and user-friendliness) into account. It is thus very possible that dose-response relationships might become more apparent when the measures used to operationalize adherence match the goal of the technology [82,83].

All of the measures for adherence in this review are based on data regarding technology usage. However, the results of a recent literature review of theoretical perspectives on adherence showed that adherence is a multidimensional concept, influenced by a range of technological, environmental, and individual factors altogether that cannot be evaluated by technology usage alone [8,84]. Therefore, additional measures are needed to determine whether and why users are or have been adherent to technology. For example, a mixed-methods approach that combines usage data with questionnaires, health measurements, and/or interviews could provide important knowledge regarding why people do or do not use the technology, how people learn from using the technology, the minimal use that is needed for users to experience certain effects or to reach certain goals, and how the skills acquired while using the technology are then applied in daily life. These outcomes could then in turn be used for determining the intended use of the technology and translating that concept into concrete measures for evaluating adherence.

In their review, Donkin et al state that it is difficult to compare adherence with different technologies when the measures that are used across the different trials vary [6]. However, this statement is based on Category A operationalizations of adherence where more use is better. When using Category B or (preferably) Category C operationalizations, the actual usage of each individual can be compared with the technology’s intended usage. In turn, the percentage of people who adhered to the intervention can be calculated, making adherence a more objective and standardized concept [1]. This approach simplifies the comparison of adherence across technologies with the same goal (eg, improving physical activity) but different technology characteristics (eg, features of the technology, persuasiveness, and user-friendliness) and a different operationalization of intended use. At the same time, this approach also simplifies the comparison of adherence of different users of a specific technology. Ideally, when individuals have different goals for using a technology, they should also have an individual intended usage, which could be used to get more fine-grained, personalized measures of adherence. This will be of added value for both developers and researchers, as this approach will provide better insight into how adherence to the various elements of different technologies contributed to the outcomes that are found, and for whom.

### Limitations

As the goal of this review was to gain insight into how the concept of adherence has been used in previous eHealth evaluations, we only included studies that used adherence as an outcome measure or studies that explicitly stated how other outcome measures are used as a proxy for adherence. As such, we may have missed relevant studies that formulated the intended use of a technology, but used other related terms for adherence (eg, nonusage attrition, engagement, drop-out, or [non]usage). In future research, added value might be obtained by reviewing these studies to find directions on how to operationalize the intended use for different eHealth technologies.

Furthermore, we defined the categories of adherence operationalizations for every study, instead of every type of technology. After all, adherence and intended use can be defined in many different ways, and it is very possible for different operationalizations to be used for different studies regarding the same technology. Even so, we feel that we have included a large body of studies in this review, providing valuable insight into the concept of adherence and intended use.

### Conclusions

Previous research has shown that users do not always have to experience all of the elements of a technology and that effective usage patterns might differ across users. However, the results of this review show that the operationalization of intended use is mostly based on the assumption of “the more use, the better” and that when a threshold of intended use is provided, justification is often missing. Therefore, it can be concluded that adherence to eHealth technology is an underdeveloped and often improperly used concept in the existing body of literature.

When the intended use of a technology was defined, the goal of the technology and/or the assumed working mechanisms often formed the starting point for selecting valid measures (eg, number of logins, number of completed modules). A justified threshold for intended use in accordance with the goal of the technology provides information for a concise evaluation of adherence and the working mechanisms of a technology. Subsequently, justified operationalization (comprising multidimensional measures or not) can be used to standardize adherence to different eHealth technologies, making it easier to compare the adherence rates of different technologies.

## Appendix

Multimedia Appendix 1 Keywords literature search.

Multimedia Appendix 2 Included studies and technologies, levels of adherence operationalization, and measures.

## Abbreviations

eHealth: electronic health
SMS: short message service
WHO: World Health Organization
CBT: cognitive behavioral therapy
