# Supplementary material for: Clarifying the Concept of Adherence to eHealth Technology: Systematic Review on When Usage Becomes Adherence
Source: J Med Internet Res. 2017 Dec 6;19(12):e402. doi: 10.2196/jmir.8578 (PMC5738543; doi:10.2196/jmir.8578)
Supplement: Multimedia Appendix 1 [file jmir_v19i12e402_app1.pdf]

Multimedia Appendix 1. Keywords literature search

Technology	Intervention	Adherence	Health
“web*page”	Intervention*	*Adheren*	Health*
“web*application”	Treatment*		Behavio*
website	Program*		Manage*
“Internet*delivered”	Therap*		“self*help”
“web*based”	Coach*		“self*control”
“internet*based”	Platform*		“self*management”
“internet*mediated”	App*		“self*care”
“internet*supported”			
online*			
"medical informatics"			
"information tech*"			
"e*health"			
"e*therap*"			
telemedic*			
Telecare			
Telehealth			
"e*mental health"			
"emental health"			
Wearable*			
M*health			
“Mobile tech*”			
“Smart*watch*”			
