# Supplementary material for: Clarifying the Concept of Adherence to eHealth Technology: Systematic Review on When Usage Becomes Adherence
Source: J Med Internet Res. 2017 Dec 6;19(12):e402. doi: 10.2196/jmir.8578 (PMC5738543; doi:10.2196/jmir.8578)
Supplement: Multimedia Appendix 2 [file jmir_v19i12e402_app2.pdf]

Multimedia Appendix 2. Included studies and technologies, levels of adherence operationalization, and measures

Author	Year	Name of technology	Healthcare area	Device	Structure	Intended usage?	Substan-tiated?	# logins/sessions	# modules/lessons	# elements	# exercises	# pages	# days/weeks/months	time spent	study-dropout	self-reported
**Alfonsson et al.**	2016	n/a	Mental	Web-based	Fixed	No	No			x	x					
**Alfonsson et al.**	2016	n/a	Mental	Web-based	Fixed	No	No			x	x					x
**Alley et al.**	2016	My Activity Coach	Lifestyle	Web-based	Fixed	Yes	No		x						x	
**An et al.**	2006	RealU	Lifestyle	Web-based	Free	Yes	No			x						
**Batterham et al.**	2008	MoodGym	Mental	Web-based	Hybrid	No	No		x							
**Beatty et al.**	2017	Finding My Way	Mental	Web-based	Hybrid	Yes	Yes	x	x			x	x	x		
**Beintner et al.**	2014	StudentBodies	Mental	Web-based	Fixed	No	No					x			x	
**Blake et al.**	2016	STAK-D	Chronic	Web-based, wearable	Free	No	No			x		x				
**Carlsen et al.**	2017	young.constant-care.com	Chronic	Web-based	Fixed	Yes	No						x			
**Carolan et al.**	2016	WorkGuru	Mental	Web-based	Hybrid	Yes	Yes	x								
**Carter et al.**	2013	MyMealMate	Lifestyle	Smartphone	Free	Yes	No						x			
**Christensen et al.**	2005	MoodGym	Mental	Web-based	Hybrid	No	No		x							
**Cruz et al.**	2014	COGWEB	Mental	Web-based	Fixed	Yes	No	x								
**El Alaoui et al.**	2015	n/a	Mental	Web-based	Fixed	No	No		x							
**Farrer et al.**	2014	BluePages (MoodGym)	Mental	Web-based	Hybrid	Yes	No		x							
**Fernandes-Taylor et al.**	2017	WoundCheck	Chronic	Smartphone	Fixed	Yes	No						x			
**Graham et al.**	2013	BecomeAnEx	Lifestyle	Web-based	Free	No	No	x			x	x		x		
**Graham et al.**	2013	BecomeAnEx	Lifestyle	Web-based	Free	No	No	x		x			x	x		

**Graham et al.**	2016	BecomeAnEx	Lifestyle	Web-based	Free	No	No	x				x		x		
**Graham et al.**	2017	BecomeAnEx	Lifestyle	Web-based	Free	No	No	x		x		x		x		
**Grossert et al.**	2016	STREAM	Mental	Web-based	Fixed	No	No	x	x	x	x			x		
**Hayman et al.**	2017	Fit4Two	Lifestyle	Web-based	Fixed	No	No		x	x						
**Ho et al.**	2014	n/a	Mental	Web-based	Fixed	No	No		x					x		x
**Jander et al.**	2016	Alcohol Alert	Lifestyle	Web-based (Game)	Fixed	No	No	x								
**Johansson et al.**	2015	n/a	Mental	Web-based	Fixed	No	No		x		x					
**Joseph et al.**	2015	n/a	Lifestyle	Web-based	Free	Yes	No	x		x	x			x		
**Kelders et al.**	2013	Living to the Full	Mental	Web-based	Hybrid	Yes	No		x							
**Kooistra et al.**	2016	n/a	Mental	Web-based (Blended)	Fixed	No	No		x							
**Lillevoll et al.**	2014	MoodGym	Mental	Web-based	Hybrid	No	No		x							
**Mananes & Vallejo**	2014	UNED	Lifestyle	Web-based	Fixed	No	No		x							
**Manwaring et al.**	2008	StudentBodies	Mental	Web-based	Free	No	No			x		x	x			
**Masse et al.**	2015	PACE	Lifestyle	Web-based	Hybrid	Yes	No			x		x	x			
**Mattila et al.**	2013	n/a	Lifestyle	Wearable, smartphone, Web-based	Free	Yes	No						x			
**McCabe et al.**	2009	Rekindle	Mental	Web-based	Fixed	No	No		x							
**Mertens et al.**	2017	Medication Plan	Chronic	Smartphone	Fixed	Yes	Yes						x			
**Mohr et al.**	2013	MoodManager	Mental	Web-based	Fixed	No	No		x	x			x			
**Murray et al.**	2013	Down Your Drink	Lifestyle	Web-based	Hybrid	No	No	x								
**Neil et al.**	2009	MoodGym	Mental	Web-based	Hybrid	No	No		x		x					
**Neve et al.**	2010	The Biggest Loser Club	Lifestyle	Web-based	Free	No	No			x			x			
**Nicholas et al.**	2010	n/a	Mental	Web-based	Fixed	Yes	No		x		x					
**Nijland et al.**	2011	DiabetesCoach	Chronic	Web-based	Free	Yes	No			x			x			
**Noone & Hogan**	2016	Headspace	Mental	Smartphone, tablet or Web-based	Hybrid	No	No		x					x		
**Nordin et al.**	2016	n/a	Lifestyle	Web-based	Free	No	No							x		
**Postel et al.**	2011	Alcohol de Baas	Lifestyle	Web-based	Fixed	Yes	No		x		x					

**Price et al.**	2013	Disaster Recovery Web	Mental	Web-based	Fixed	No	No		x							
**Price et al.**	2012	Disaster Recovery Web	Mental	Web-based	Fixed	No	No		x							
**Reinwand et al.**	2015	n/a	Lifestyle	Web-based	Fixed	Yes	Yes		x							
**Richardson et al.**	2010	Stepping Up to Health	Lifestyle	Wearables, Web-based	Hybrid	Yes	No						x			
**Rodgers et al.**	2016	n/a	Lifestyle	Smartphone	Fixed	Yes	No						x			
**Rutledge et al.**	2017	TeleMOVE	Lifestyle	Monitor, digital scale, pedometer	Fixed	Yes	No						x			
**Schultz et al.**	2016	n/a	Mental	Web-based	Hybrid	No	No		x	x				x		
**Schuster et al.**	2017	n/a	Mental	Web-based	Fixed	Yes	No		x							
**Titov et al.**	2013	Wellbeing Course	Mental	Web-based	Fixed	Yes	No		x							
**Tu et al.**	2017	MySteps	Lifestyle	Web-based	Fixed	No	No					x	x			
**Van den Berg et al.**	2013	BREATH	Mental	Web-based	Free	Yes	No	x					x			
**Van der Zanden et al.**	2014	Master Your Mood	Mental	Web-based	Fixed	Yes	No						x			
**Wang et al.**	2016	My Trauma Recovery	Mental	Web-based	Hybrid	No	No		x			x	x			
**Wangberg et al.**	2008	n/a	Chronic	Web-based	Free	Yes	No	x						x		
**Wanner et al.**	2010	Active-online	Lifestyle	Web-based	Free	No	No		x			x		x		
**Ware et al.**	2008	MiLife	Lifestyle	Web-based, wearable, weighing scale	Free	No	No	x						x		
**Zarski et al.**	2016	GET.ON Stress	Mental	Web-based	Hybrid	Yes	No		x							
**Zeng et al.**	2016	n/a	Lifestyle	Smartphone	Free	Yes	Yes		x	x						
